# New Insight into the Time-Course of Motor and Sensory System Changes in Pain

**DOI:** 10.1371/journal.pone.0142857

**Published:** 2015-11-24

**Authors:** Siobhan M. Schabrun, Emma Burns, Paul W. Hodges

**Affiliations:** 1 Western Sydney University, Brain Rehabilitation and Neuroplasticity Unit, School of Science and Health, Campbelltown Campus, Locked Bag 1797, Penrith NSW 2751, Australia; 2 The University of Queensland, NHMRC Centre of Clinical Research Excellence in Spinal Pain, Injury and Health, School of Health and Rehabilitation Science, St Lucia, Brisbane, Queensland 4062, Australia; University Medical Center Goettingen, GERMANY

## Abstract

**Background:**

Pain-related interactions between primary motor (M1) and primary sensory (S1) cortex are poorly understood. In particular, the time-course over which S1 processing and corticomotor output are altered in association with muscle pain is unclear. We aimed to examine the temporal profile of altered processing in S1 and altered corticomotor output with finer temporal resolution than has been used previously.

**Methods:**

In 10 healthy individuals we recorded somatosensory evoked potentials (SEPs) and motor evoked potentials (MEPs) in separate sessions at multiple time-points before, during and immediately after pain induced by hypertonic saline infusion in a hand muscle, and at 15 and 25 minutes follow-up.

**Results:**

Participants reported an average pain intensity that was less in the session where SEPs were recorded (SEPs: 4.0±1.6; MEPs: 4.9±2.3). In addition, the time taken for pain to return to zero once infusion of hypertonic saline ceased was less for participants in the SEP session (SEPs: 4.7±3.8 mins; MEPs 9.4±7.4 mins). Both SEPs and MEPs began to reduce almost immediately after pain reached 5/10 following hypertonic saline injection and were significantly reduced from baseline by the second (SEPs) and third (MEPs) recording blocks during pain. Both parameters remained suppressed immediately after pain had resolved and at 15 and 25 minutes after the resolution of pain.

**Conclusions:**

These data suggest S1 processing and corticomotor output may be co-modulated in association with muscle pain. Interestingly, this is in contrast to previous observations. This discrepancy may best be explained by an effect of the SEP test stimulus on the corticomotor pathway. This novel finding is critical to consider in experimental design and may be potentially useful to consider as an intervention for the management of pain.

## Introduction

Pain alters motor and sensory function. Yet, pain-related interactions between the primary motor (M1) and primary sensory (S1) cortices are poorly understood. Most authors agree S1 excitability, measured using somatosensory evoked potentials (SEPs) to noxious/non-noxious peripheral electrical stimuli, and corticomotor output, measured using motor evoked potentials (MEPs) to transcranial magnetic stimulation (TMS), are reduced in response to pain [1–5]. However, the time-course over which S1 processing and corticomotor output are altered, and the temporal relationship between them, is less clear. This information is essential to understand the mechanisms that underpin altered sensorimotor function in pain.

Only one study has examined the temporal relationship between altered activity in S1 and corticomotor output in response to acute muscle pain. That study demonstrated altered S1 processing (during pain) prior to reduced corticomotor output (present once pain had resolved) [5]. This time-course suggests that either S1 and M1 excitability are modified by independent processes in the presence of pain, or that altered S1 processing during acute muscle pain mediates a latent reduction in motor output. A key limitation of that study is that the temporal relationship between S1 and M1 was measured only at three distinct time points (before, during and after pain), limiting interpretation of the evolution of these changes over time. Examination of processing in these cortical regions using greater temporal resolution is required to refine hypotheses regarding sensory-motor cortical interactions in response to acute muscle pain. Here we aimed to investigate the temporal profile of altered processing in S1 and altered corticomotor output at multiple time-points before, during and immediately after pain in a hand muscle, and at 15 and 25 minutes follow-up.

## Experimental Procedures

### Participants

Ten right-handed healthy individuals (6 males, 4 females; mean ± standard deviation 22 ± 2 years) participated in two experimental sessions (one to examine sensory cortex excitability and the other to examine corticomotor output) on separate days. Session order was randomised. Sample size was determined based on MEP and SEP data obtained before and after hypertonic saline infusion from a previous study (MEP: minimum detectable difference in means of 0.5mV and standard deviation of 0.3 mV; SEP: minimum detectable difference in means of 3.8μV^2^ and standard deviation of 2.7μV^2^) [5]. Using these values we required 7 participants for MEPs and 9 participants for SEPs with a power of 80% and an alpha of 0.05 to detect an effect of pain over time, should one exist. Participants had no history of upper limb or neurological conditions and completed a TMS safety screen prior to commencement. Experimental protocols were approved by The University of Queensland’s Human Medical Research Ethics Committee and participants gave written informed consent. All procedures were conducted in accordance with the declaration of Helsinki.

### Electromyography

Electromyography (EMG) was recorded with surface electrodes from the first dorsal interosseous (FDI) muscle of the right hand. Disposable silver/silver chloride adhesive recording electrodes (Noraxon USA Inc, Arizona, USA) were positioned over the muscle belly approximately in parallel with muscle fibres. The ground electrode was placed over the right ulnar styloid process. Signals were amplified 1000x, band pass filtered between 20–1000 Hz and sampled at 2000 Hz using a Micro 1401 data acquisition system and Signal software (CED Limited, Cambridge, UK).

### Transcranial magnetic stimulation

Transcranial magnetic stimulation was delivered using a Magstim 200 stimulator (Magstim Co Ltd, Dyfed, UK) and a figure-of-eight coil (7 cm external wing diameter). The coil was positioned over the left hemisphere and orientated at a 45° angle to the sagittal midline to preferentially induce current in a posterior-to-anterior direction. The optimal cortical site to evoke responses in right FDI was determined as the coil position that evoked the largest motor evoked potential (MEP; quantified as peak-to-peak amplitude) in the contralateral FDI muscle. Stimulus intensity was adjusted to produce a MEP of approximately 1 mV in relaxed FDI at baseline and this intensity was kept constant throughout the experiment. Participants were instructed to keep their hand relaxed throughout the experiment. All TMS procedures adhered to guidelines for methodological quality [6].

### Ulnar nerve stimulation

To record M-waves, single electrical stimuli (duration: 200 μs and max current of 1 A) were delivered to the right ulnar nerve at the wrist using a constant current stimulator (DS7A, Digitimer Ltd, Welwyn Garden City, UK). Intensity was set 50% above that needed to evoke a maximal compound muscle action potential in FDI EMG.

### Electroencephalography (EEG)

SEPs were recorded in response to stimulation of the right ulnar nerve at the wrist. EEG was recorded from the approximate location of the hand area of the primary sensory cortex using gold plated cup electrodes positioned over C3’ (2 cm posterior to C3) and referenced to Fz. Additional electrodes were placed over the cervical spine (C7) and Erb’s point to track the afferent volley in the spinal cord and peripheral nerve, respectively. Electrode impedance was maintained below 5 kΩ. EEG signals were amplified 50000x, band pass filtered between 5–500 Hz and sampled at 1000 Hz using the same system as the EMG recordings.

A constant current stimulator was used to deliver electrical stimuli of 1-ms duration to the ulnar nerve at a rate of 2/s (maximum current: 1 A). A 20% variance was incorporated into the stimulus frequency to avoid accommodation (range 1.6 to 2.4 Hz). Perceptual threshold was determined using the method of limits, averaging over the last two of three ascending and descending series. Stimulus intensity was set at 3x perceptual threshold and kept constant throughout the experiment. This intensity was considered comfortable by all participants and was sufficient to evoke a visible muscle twitch in FDI. Each block consisted of 500 stimuli that were averaged off-line for analysis.

### Experimental muscle pain

Experimental muscle pain was induced by infusion of 5% hypertonic saline into right FDI using a syringe pump (ALARIS Medical Systems Australia, Australia). A 22-gauge disposable cannula was inserted into right FDI with the tip at a depth of ~0.5 cm. The cannula was connected to a 10 ml plastic syringe by a low sorbing extension tube (IVAC Medical Systems, UK). A single bolus of 0.2 ml of sterile hypertonic saline was infused over 20 s, followed by a steady infusion rate of 6 ml/h. This experimental pain paradigm has been shown to induce a rapid increase in pain within the first 60 s (reaching peak pain within this timeframe) before producing a relatively consistent pain rating of moderate intensity [7].

Pain intensity was rated at the beginning of each MEP and SEP recording block using an 11-point numerical rating scale (NRS) anchored with “no pain” at 0 and “worst pain imaginable” at 10. At the conclusion of the experiment, participants completed the Short Form McGill Pain Questionnaire (MPQ) [8].

### Experiment 1—Corticomotor output

Participants sat comfortably with their right hand relaxed. Following cannula insertion, time was allowed for any localised pain to return to resolve completely before baseline measures were recorded. Three blocks of 12 MEPs were recorded prior to the induction of experimental pain to ensure a stable baseline. Once pain intensity was reported as 5/10 on the NRS after injection of hypertonic saline, a further 5 blocks of 12 MEPS were recorded. The syringe pump was then turned off and 1 block of 12 MEPs recorded as pain returned towards zero. Two blocks of 12 MEPS were recorded at 15 and 25 minutes after pain had returned to zero. Four M-waves were recorded at each time point (baseline, during pain, pain return to zero, post pain 1 and post pain 2). MEPs were recorded at a rate of one every 15 seconds with a 2.16 minute rest interval applied between each block (total time to record one block 5.16 minutes). Following the induction of pain, a timer was used to ensure that recording of each subsequent MEP and SEP block was consistent between the two experiments.

### Experiment 2 –S1 processing

**Fig 1:**
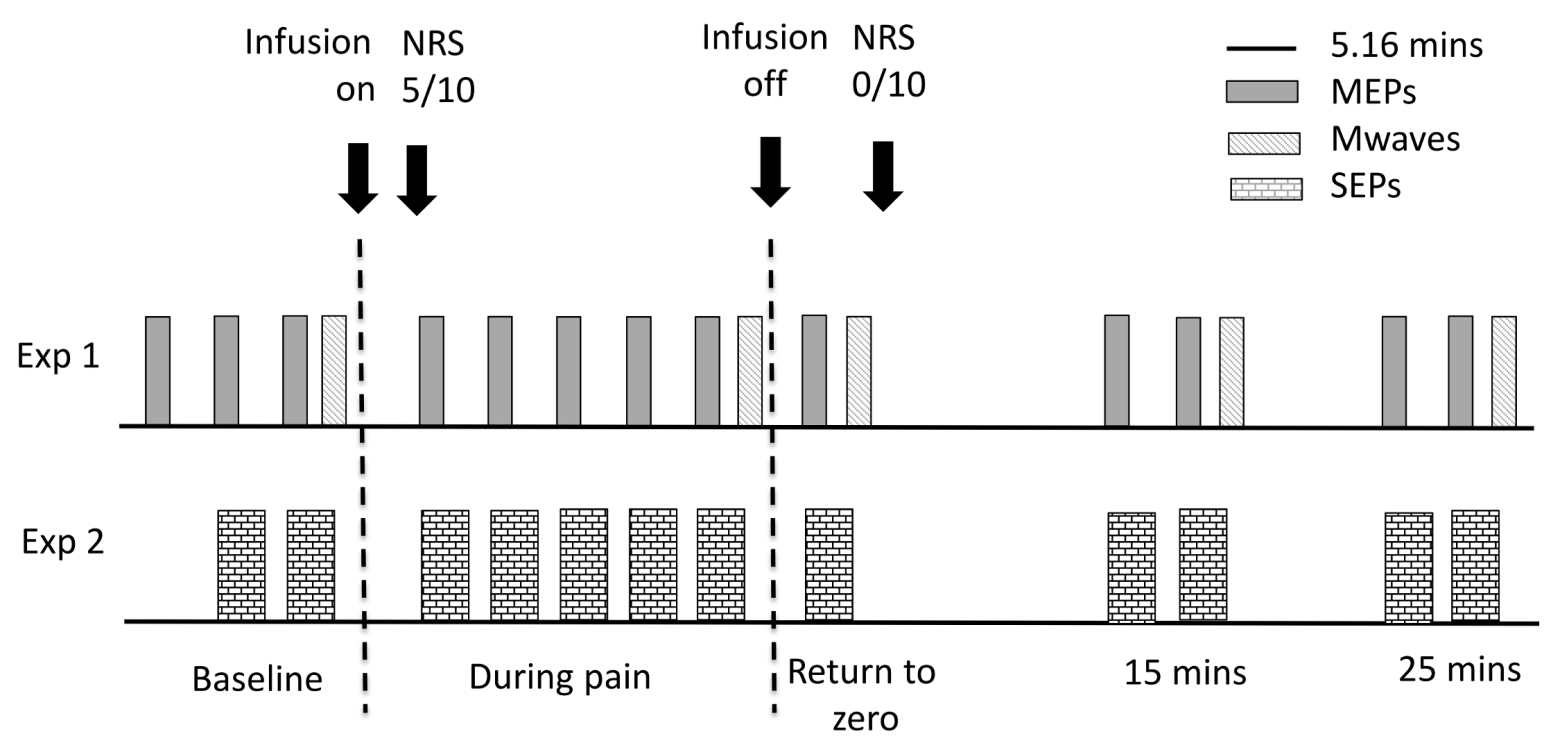
Protocol and timing of measurements for Experiments 1 and 2. A stopwatch was used to ensure each block of MEPs and SEPs was recorded at the same time across the two experiments.

Set up was identical to Experiment 1. Two blocks of 500 SEPs were recorded at baseline, followed by induction of experimental pain. Once pain intensity was reported as 5/10 on the NRS a further 5 blocks of SEPs were recorded. The syringe pump was then turned off and one block of SEPS recorded as pain returned to zero. Two further blocks of SEPs were recorded 15 and 25 minutes after pain had returned to zero. A one-minute rest interval was applied between each SEP block (total time to record one block 5.16 minutes). Protocols for experiments 1 and 2 are summarised in Fig 1.

### Experiment 3 –corticomotor output and S1 processing in response to a non-painful injection of isotonic saline as a control

Seven healthy individuals (3 males, 4 females; mean and standard deviation age of 26 ± 4 years) participated in two experimental sessions to examine sensory cortex excitability and corticomotor output in response to a non-painful control injection. All procedures were identical to those described for Experiments 1 and 2 above, except that isotonic saline (0.2 ml, 0.9%) was injected into the cannula following recording of the baseline measures.

### Data and statistical analysis

**Fig 2:**
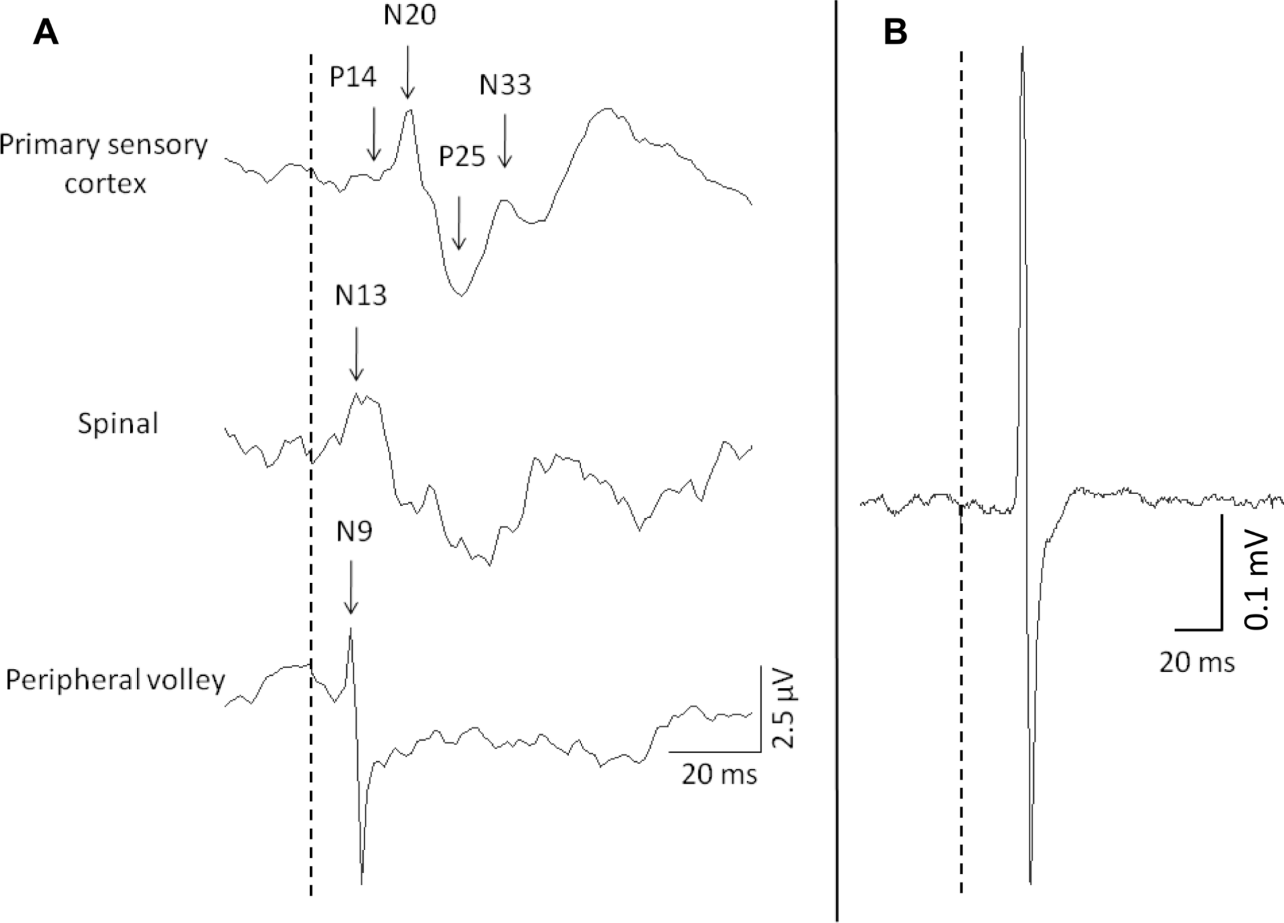
Raw data from a single participant demonstrating A) the SEP components used for analysis at the primary sensory cortex, spinal cord and the peripheral volley recorded at Erb’s point and B) a MEP trace. The dotted line represents the time of stimulation in both panels.

Peak-to-peak amplitudes of MEPs and M-waves were measured and averaged across trials within each set. MEP data from the three blocks recorded at baseline (pre-pain) were averaged to create a single baseline value. Similarly, data from the two 15-minute follow-up blocks and two 25-minute follow-up blocks were averaged to create a single value at each time-point. Blocks that were averaged demonstrated no statistical difference prior to averaging. Data were analysed separately for the pain and control conditions using a one-way repeated measures analysis of variance (ANOVA) with factor block (pre/during/pain return to zero/15 min post pain/25 min post pain). SEPs were analysed as peak-to-peak amplitudes for the biphasic peripheral (N_9_), spinal (N_13_), subcortical (P_14_-N_20_) and cortical (N_20_-P_25_; P_25_-N_33_) waves, and as area for the N_20_-P_25_-N_33_ complex. Data from the two blocks recorded at baseline (pre-pain), two 15-minute follow-up blocks and two 25-minute follow-up blocks were averaged to create a single value at each of these time-points. Onset latencies were calculated as time from stimulus onset to peak N_9_, N_13_, N_20_, P_25_, and N_33_ volleys. Amplitudes, areas and latencies were compared between blocks (pre/during/return to zero/15 min post pain/25 min post pain) using separate one-way repeated measures ANOVA for each component in the pain and control conditions. Examples of SEP and MEP traces and the components that were analysed are provided in Fig 2.

Average pain intensity during the hypertonic saline infusion and the time taken for pain to return to zero once the hypertonic saline infusion was ceased were compared between the two experiments using one-way repeated measures ANOVA. Where appropriate, post-hoc tests were performed using Holm-Sidak multiple comparison tests. The Holm-Sidak method is able to correct for Type 1 errors as effectively as the traditional Bonferroni method while retaining greater statistical power [9]. Significance was set at 5%. Data are presented as mean ± standard deviation throughout the text.

Percent change scores were calculated at each time-point relative to baseline (e.g. ((During-Baseline/Baseline)*100)) and linear regression analyses performed to determine whether S1 excitability was associated with corticomotor excitability in response to pain. Linear regression analyses were also performed to determine whether the average percent change in S1 excitability and corticomotor output during pain was associated with pain severity.

## Results

### Pain Characteristics

Average pain intensity reported during infusion of hypertonic saline differed between Experiments 1 and 2 (F_49,49_ = 7.3, p = 0.01), with less pain experienced when SEPs were recorded (4.0±1.6) than when MEPs were recorded (4.9±2.3). The words most commonly selected to describe the pain for both protocols were “aching” (82%), “throbbing” (64%) and “cramping” (45%). All participants reported symptoms localized to the infusion site and radiating toward the thumb and index finger. In addition, the time taken for pain to return to zero once the hypertonic saline infusion was ceased differed between Experiments 1 and 2 (F_9,9_ = 8.0, p = 0.02). When SEPs were recorded, pain returned to zero in 4.7±3.8 minutes, but when MEPs were recorded pain returned to zero in 9.4±7.4 minutes. The injection of isotonic saline did not produce a sensation of pain in any individual, with all participants scoring 0/10 on the NRS in both the MEP and SEP recording sessions in Experiment 3.

### Experiment 1. MEPs are suppressed during and after pain

**Fig 3:**
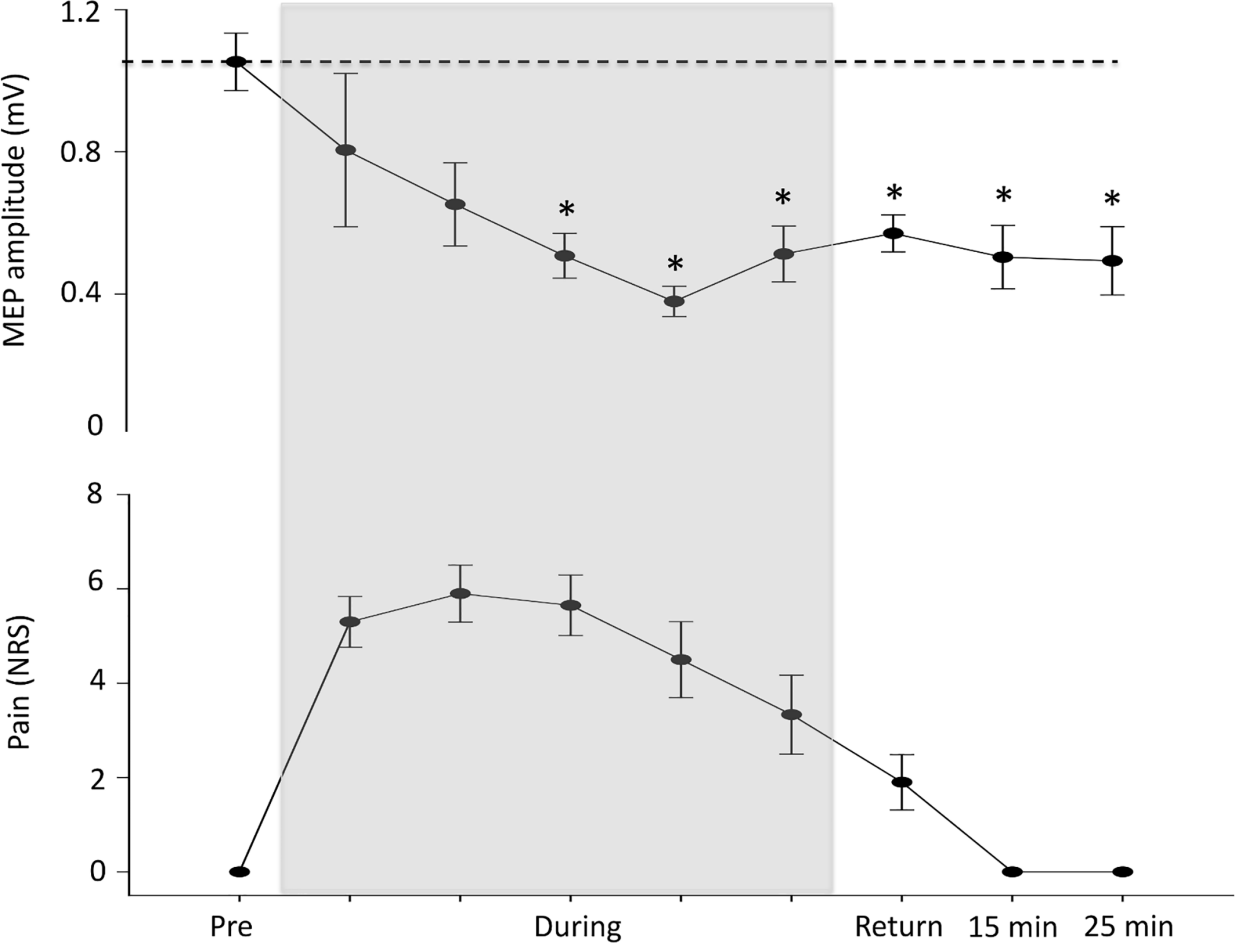
Group data (mean ± SE) from experiment 1 demonstrating the motor evoked potential (MEP) amplitude (upper graph) and pain (numerical rating scale; NRS; lower graph) at each time point before, during and after the hypertonic saline infusion. The dotted line represents baseline and the shaded area the duration of the hypertonic saline infusion. The amplitude of the MEP was suppressed at block 3 during the painful infusion and remained suppressed at 25 minutes follow-up. *—P<0.05.

The average stimulator output required to obtain a MEP of approximately 1 mV peak-to-peak at baseline was 57±13%. There was no difference in the amplitude of the MEP between the three blocks (block 1: 1.12±0.3 mV, block 2: 1.07±0.31 mV, block 3: 0.96±0.30 mV) recorded at baseline (F_9,18_ = 1.6, p = 0.24). The induction of pain in right FDI suppressed MEP amplitudes (main effect of block F_9,72_ = 4.3, p <0.001). MEP amplitude was reduced by the third block of MEPs recorded during the pain infusion (post hoc—pre vs. block 1: p = 0.88; pre vs. block 2: p = 0.14; pre vs. block 3: p = 0.006; pre vs. block 4: p<0.001; pre vs. block 5: p = 0.045) and did not recover to baseline values by the 25 min follow-up (pre vs. pain recovery p = 0.025; pre vs.15 min p = 0.007; pre vs. 25 min p = 0.002; Fig 3). M-wave amplitude did not change over time (F_9,36_ = 0.42, p = 0.79), which excludes any effects of the protocol at the muscle and other peripheral elements of the motor pathway. There was no relationship between the size of the reduction in corticomotor output and pain severity (r^2^ = 0.0005, p = 0.95) during pain.

### Experiment 2. SEPs are suppressed during and after pain

**Fig 4:**
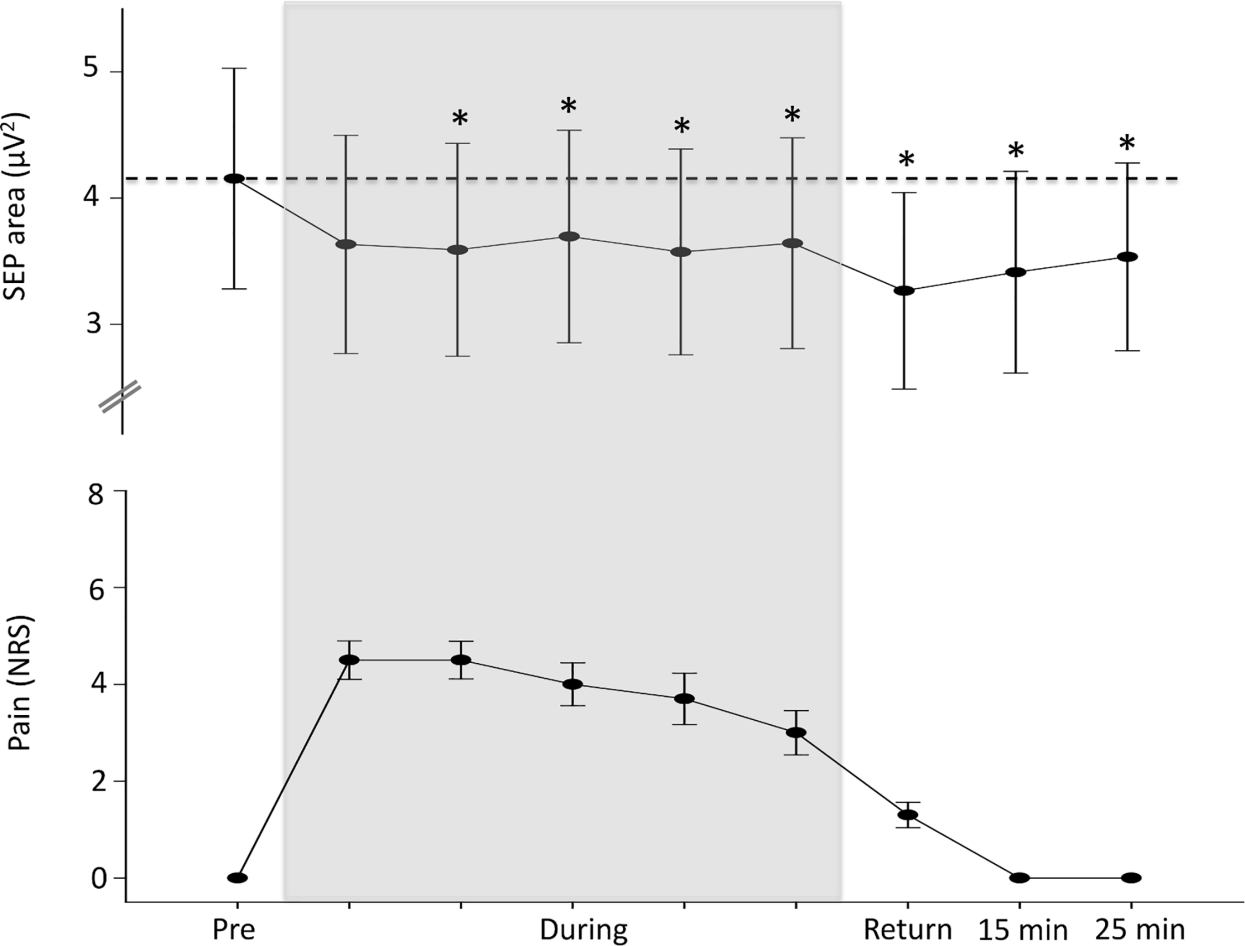
Group data (mean ± SE) from experiment 2 demonstrating the area of the SEP N_20_–P_25_–N_33_ complex (upper graph) and pain (numerical rating scale; NRS; lower graph) at each time point before, during and after the hypertonic saline infusion. The dotted line represents baseline and the shaded area the duration of the hypertonic saline infusion. The area of the SEP was suppressed at block 2 during the painful infusion and remained suppressed at 25 minutes follow-up. *—P<0.05.

**Fig 5:**
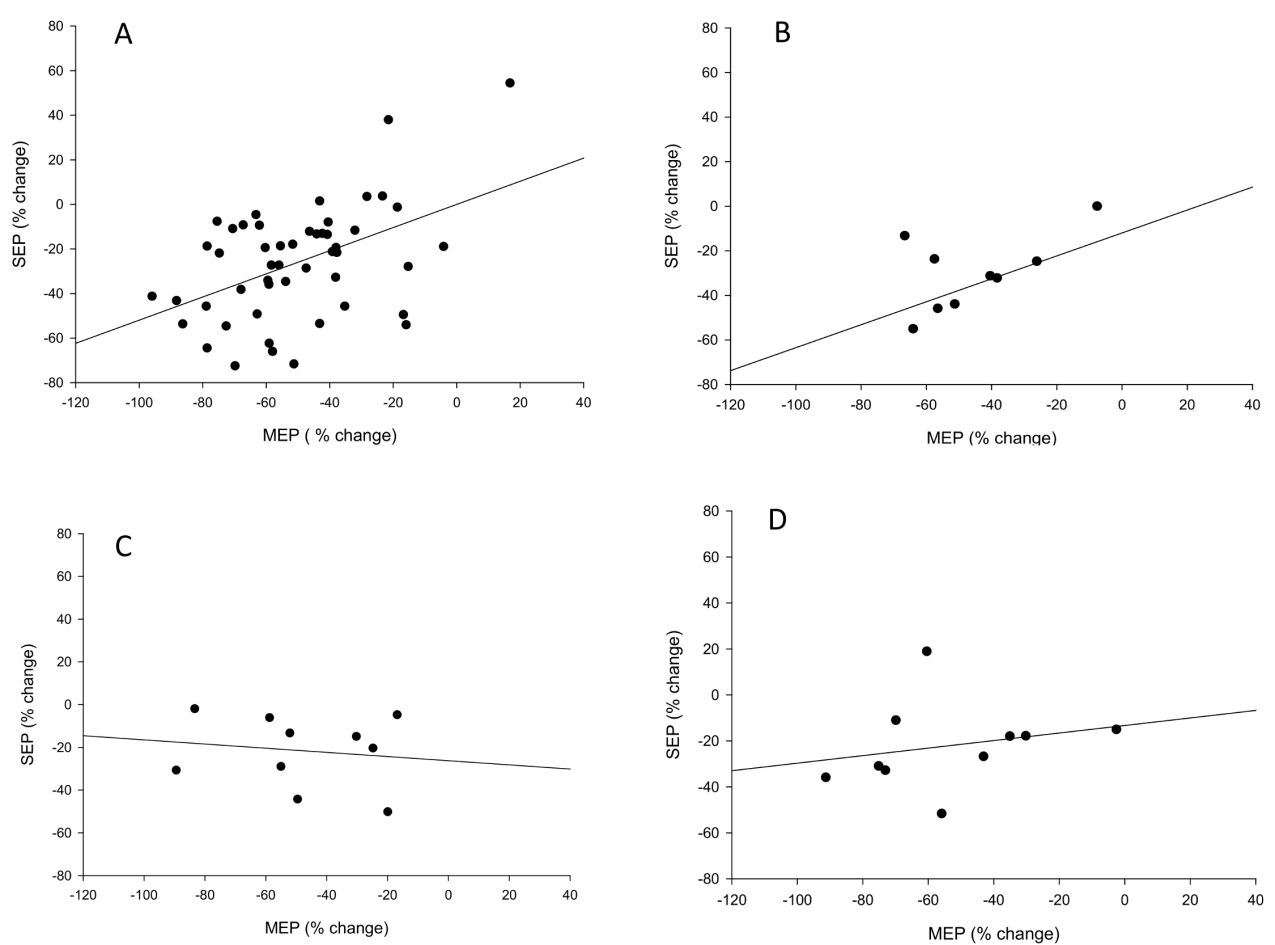
Linear regression of per cent change scores for SEPs and MEPs relative to baseline A) during pain (across the 5 recording blocks), B) as pain returned to zero, C) 15 minutes and D) 25 minutes after pain had resolved. A larger reduction in the size of the SEP was associated with a larger reduction in the size of the MEP when pain was present (P<0.001, r^2^ = 0.22).

At three times perceptual threshold, the average intensity of the electrical stimulation at baseline was 11.2±2.4 mA. There was no difference between the two blocks of SEPs recorded at baseline (SEP complex area block 1: 4.0±2.8μV^2^, block 2: 4.2±2.8μV^2^; F_9,9_ = 0.67, p = 0.44). The size and latency of the peripheral (effect of block F_9,72_ = 0.6, p = 0.77 amplitude; F_9,72_ = 0.76, p = 0.55 latency) and spinal (effect of block F_9,72_ = 1.7, p = 0.10 amplitude; F_9,72_ = 0.94, p = 0.45 latency) complexes were consistent across time. This confirms stability of the input to the sensory cortex throughout the protocol. Infusion of hypertonic saline induced a reduction in the area of the N_20_-P_25_-N_33_ complex (effect of block F_9,72_ = 4.8, p<0.001). Suppression of the SEP complex was evident by block 2 during the pain infusion (post hoc—pre vs. block 1: p = 0.1; pre vs. block 2: p = 0.024; pre vs. block 3: p = 0.019; pre vs. block 4: p = 0.002; pre vs. block 5: p = 0.002). This effect persisted once pain had recovered (p<0.001) and at the 15- (p = 0.025) and 25-minute (p = 0.003) follow up (Fig 4). The amplitude of the N_20_-P_25_ component also reduced (effect of block F_9,72_ = 15.8, p<0.001). This effect was present at all time-points during the pain infusion (all p<0.001) and as pain recovered (p<0.001) but did not persist at the 15- (p = 0.84) or 25-minute follow-up (p = 0.77). A larger reduction in the magnitude of the SEP was associated with a larger reduction in the MEP during pain (p<0.001, r^2^ = 0.22; Fig 5A). Similar associations were not observed as pain returned to zero (p = 0.25, r^2^ = 0.16; Fig 5B) or at 15 (p = 0.68, r^2^ = 0.02; Fig 5C) and 25 (p = 0.52, r^2^ = 0.05; Fig 5D) minutes following the resolution of pain. There was no relationship between the size of the reduction in S1 excitability and pain severity (r^2^ = 0.002, p = 0.90) during pain.

No changes in latencies (effect of block N_20_ : F_9,72_ = 0.49, p = 0.74; P_25_ : F_9,72_ = 1.0, p = 0.41; N_33_: F_9,72_ = 1.8, p = 0.16) or amplitudes (effect of block P_14_-N_20_: F_9,72_ = 0.53, p = 0.83; P_25_-N_33_: F_9,72_ = 1.3, p = 0.24) were present for any other component (S1 Table).

### Experiment 3. MEPs and SEPs are unaltered in response to a non-painful injection of isotonic saline as a control

The average stimulator output required to obtain a MEP of approximately 1 mV peak-to-peak at baseline was 49±10%. Motor evoked potential (F_8,48_ = 0.56, p = 0.80) and M-wave (F_4,24_ = 0.94, p = 0.46) amplitudes were unaltered over time in response to injection of isotonic saline (S1 Fig). At three times perceptual threshold, the average intensity of the electrical stimulation used to elicit a SEP at baseline was 13.1±4.9 mA. Injection of isotonic saline did not alter the area of the N_20_-P_25_-N_33_ complex (F_8,48_ = 1.0, p = 0.45), and there was no change in latencies (all F_8,48_<1.13, p>0.36) or amplitudes (F_8,48_<1.27, p>0.28) for any other component (S2 Fig).

## Discussion

This study aimed to investigate the time-course of altered processing in S1 and corticomotor output in association with acute muscle pain with finer temporal resolution than has been used previously. Unexpectedly this study provides evidence of a similar temporal profile of altered processing in S1 and reduced corticomotor output in response to muscle pain. This novel observation has two major outcomes. First, these data clarify a key aspect of the potential interaction between sensory and motor systems in acute muscle pain. Second, the failure to replicate earlier observations of the temporal displacement between sensory and motor effects is best explained by an effect of the SEP test stimulus on the corticomotor pathway. This finding is critical to consider in experimental design and suggests that electrical stimulation of similar intensity and frequency to that of a SEP test stimulus may be useful to consider as a clinical intervention to reduce pain severity and duration.

### Similar temporal profile of S1 processing and corticomotor output in acute muscle pain

This study is the first to demonstrate a similar temporal profile of reduction in S1 processing and corticomotor output in association with acute muscle pain. Both parameters began to reduce almost immediately after pain reached 5/10 on the NRS following hypertonic saline injection and were significantly reduced from baseline by the second (SEPs) or third (MEPs) recording blocks during pain. Both parameters remained suppressed immediately after pain had resolved and at 15 and 25 minutes after the resolution of pain. The magnitude of the reduction in S1 excitability was positively correlated with the magnitude of the reduction in corticomotor output during acute muscle pain. Although, it is unclear why altered processing in S1 and reduced corticomotor output persist following the resolution of pain, one possibility is that altered S1 and M1 activity persist until the threat that movement may evoke pain has completely resolved. An alternate possibility is that pain may motivate the nervous system to change behaviour, but recovery from pain may not necessarily motivate recovery of nervous system change. These hypotheses require further investigation. Notably, pain severity was not associated with the magnitude of the reduction in S1 excitability or corticomotor output, suggesting that the presence of pain, regardless of severity, is sufficient to drive alterations in these parameters. It is not surprising or uncommon for objective measures of physiological parameters to not be correlated with pain. This is because pain severity is a subject feature and depends on many factors such as the threat value of the stimulus and an individual’s previous experience with pain.

Although the findings for S1 excitability are consistent with those reported previously [5], the time-course of reduced corticomotor output differed. Here we observed reduced corticomotor output both during and after pain, which contrasts earlier observations that MEPs were only reduced after the resolution of pain [5]. The reduction in corticomotor output after but not during pain in the previous study led to the suggestion that depression of S1 excitability (present both during and after pain) precedes reduced corticomotor output [5]. This observation was interpreted to suggest that either; altered excitability of S1 and M1 represent independent processes in the presence of pain, or that reduced S1 excitability during pain mediates a latent reduction in motor output via processes that are non-linear [5]. However, the present data challenge these interpretations and provide evidence of a similar temporal profile for S1 excitability and corticomotor output. A possible mechanism for this observation is that nociceptive information is relayed to S1 via thalamo-cortical projections, activating a reduction in S1 processing that provides the signal (via cortico-cortical projections) for reduced corticomotor output. Cortico-cortical projections have been identified between S1 and M1 in animals and humans [10, 11] and stimulation of S1 can induce long-term potentiation of motor cortical synapses, probably through altered discharge of intracortical interneurons [12]. As changes in intracortical networks have been identified in acute muscle pain [13], this mechanism may underpin the co-modulation of S1 and M1 observed here. An alternate possibility is that direct connections between the thalamic nucleus and M1 [14–16] result in information being relayed simultaneously to S1 and M1, providing the stimulus for reduced excitability in both cortical regions within a similar timeframe. Further work is needed to disentangle the contribution of these pathways to the pain-evoked cortical response.

### Recording of SEPs may prevent, reduce or mask altered corticomotor output during pain

The differing time-course of the depression in corticomotor output in this study and that reported previously [5], is likely explained by a major difference in methodology between studies—in the previous study SEPs and MEPs were recorded in the same experimental session [5]. The difference in outcome reveals an important observation with both experimental and potential clinical significance. As all other aspects of the experimental protocol were similar, one interpretation is that electrical nerve stimulation used to measure S1 processing interfered with the reduction in corticomotor output during, but not after, muscle pain.

Several mechanisms may explain the ability of electrical stimulation to prevent, reduce or mask altered corticomotor output during pain. First, afferent input is known to be a powerful driver of cortical reorganisation. Peripheral electrical nerve stimulation, a form of repeated afferent input, can alter sensory and motor cortical excitability in a manner reminiscent of neuroplastic (long-term potentiation and long-term depression-like) mechanisms [17]. Whether cortical excitability is enhanced or supressed by peripheral electrical stimulation depends on the intensity [18, 19], frequency [18, 20] and in the case of the primary motor cortex, duration [21] of electrical stimulation. Although the effects of the specific electrical stimulation paradigm used to record SEPs (frequency of 2 Hz, intensity sufficient to induce a visible motor twitch) on the motor cortex have not been investigated, it is possible that electrical nerve stimulation masked the reduction of corticomotor during pain in the previous study via an effect on intracortical neuronal networks [13] a reduction in intracortical facilitation (ICF), thought to be mediated by NMDA receptor activity on glutamatergic interneurons [22], that is present during and after pain and an increase in short-interval intracortical inhibition (SICI), mediated by GABA_A_ inhibitory pathways [23, 24], that is present only once pain has resolved. One explanation for the discrepancy between the current and previous studies [5] is that electrical nerve stimulation influenced these intracortical neuronal networks, abolishing the reduction in ICF, and thus the reduction in corticomotor output, during pain. In the post-pain period, electrical nerve stimulation may be insufficient to overcome both the reduction in ICF and the increase in SICI. Previous work has shown that depression of MEP amplitudes is more profound after peak-pain than during [1]. These data suggest electrical nerve stimulation may prevent, reduce or mask altered corticomotor output during pain and there is potential that this phenomenon may be exploited clinically. However, it is unclear how corticomotor output and altered activity in intracortical networks relates to motor function as there is limited evidence of motor effects that share a similar time-course, particularly when a short-lasting experimental pain model is used. Future studies should seek to investigate the relationship between motor and sensory measures and motor function using a range of pain models to further clarify this issue.

An alternative possibility is that electrical nerve stimulation increased motoneuron excitability, masking any reduction in excitability from M1 during pain. One feature of peripheral electrical stimulation is that it actives nerve fibres both orthodromically and antidromically. The antidromic impulse can depolarise the anterior horn cell and potentially induce long-term potentiation and/or increased motoneuron excitability [25]. Similarly, afferent input generated by the muscle twitch evoked by the frequency and intensity used to record SEPs (2Hz, three times perceptual threshold) may have increased motoneuron excitability. We have previously shown that peripheral electrical stimulation at a frequency and intensity sufficient to evoke only a motor twitch (10 Hz, visible motor twitch) and not a ramped, tetanic motor contraction, does not alter excitability of the corticomotor pathway [19]. However, the effect of 2 Hz stimulation protocols on motoneuron and cortical excitability is unknown. Further research is needed to determine whether peripheral electrical stimulation prevents, reduces or masks a reduction in corticomotor output through spinal or cortical mechanisms.

### Research and clinical implications

These data have implications for studies that record corticomotor output while simultaneously assessing S1 processing and suggest that these aspects of cortical function should be evaluated in different experimental sessions. Notably, peripheral electrical stimulation used to record SEPs not only interfered with a reduction in corticomotor output during pain but also lowered average pain intensity and the time taken for pain to return to zero following cessation of the hypertonic saline infusion. Taken together, these unique findings suggest peripheral electrical stimulation applied at low frequency and moderate intensity may have the potential to be exploited clinically for the treatment of motor dysfunction observed in acute and chronic pain conditions, in addition to its effect on pain intensity and duration [26]. However, as this was an incidental finding from the current study, this concept requires further investigation using appropriate study designs.

## Supporting Information

S1 Fig Group data (mean ± SE) from experiment 3 demonstrating the motor evoked potential (MEP) amplitude at each time point before, during and after administration of isotonic saline. There was no change in the amplitude of the MEP over time (P>0.05).
(TIF)

S2 Fig Group data (mean ± SE) from experiment 3 demonstrating the area of the SEP N_20_–P_25_–N_33_ complex at each time point before, during and after administration of isotonic saline. There was no change in the area of the SEP N_20_–P_25_–N_33_ complex over time (P>0.05).
(TIF)

S1 Table Group data (mean and standard deviation) for amplitude and latencies of each SEP component in response to hypertonic saline infusion. (PDF)
