# Supplementary figures and images for: New Insight into the Time-Course of Motor and Sensory System Changes in Pain
Source: PLoS One. 2015 Nov 24;10(11):e0142857. doi: 10.1371/journal.pone.0142857 (PMC4658023; doi:10.1371/journal.pone.0142857)

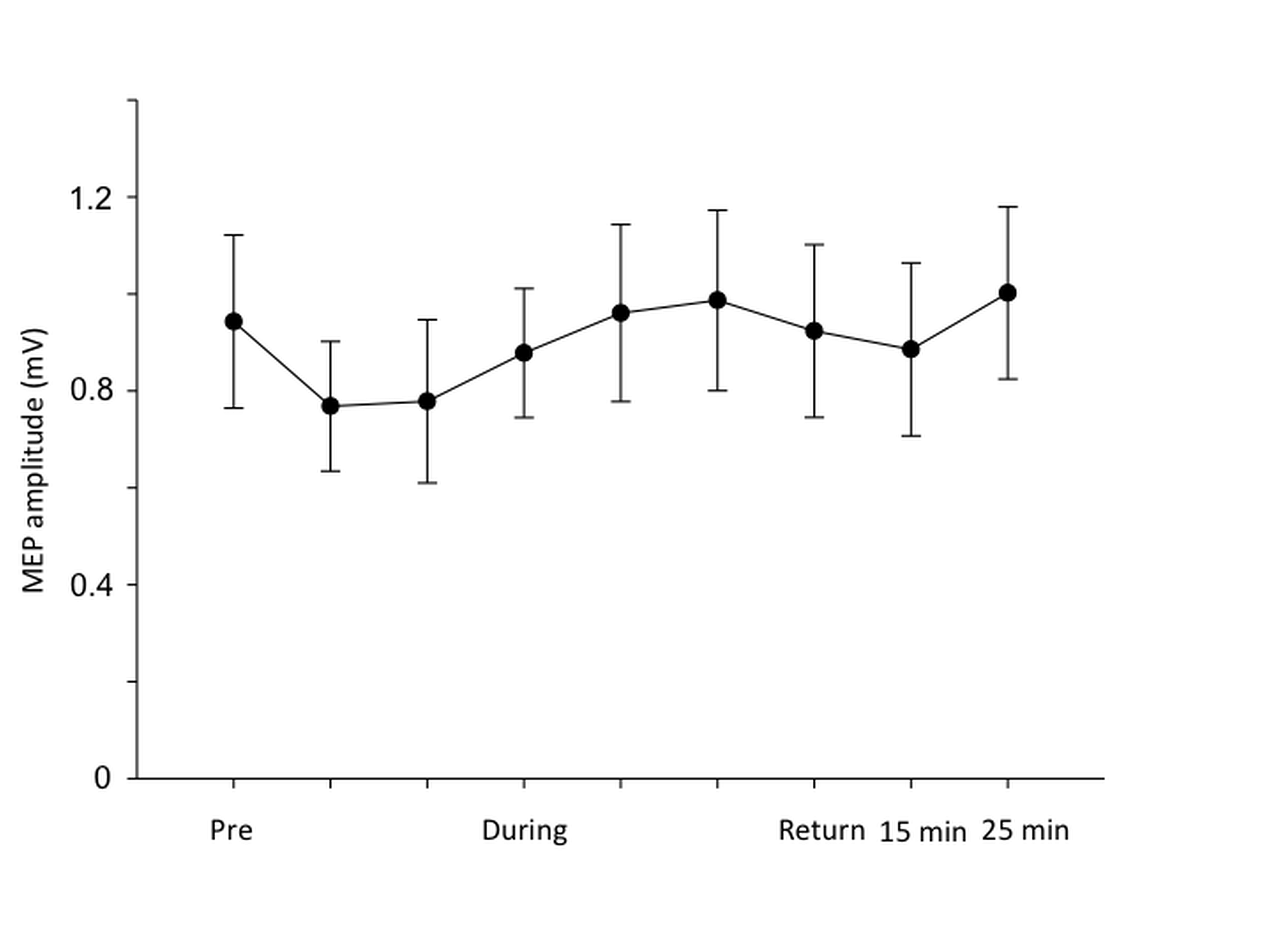

Supplement: S1 Fig — There was no change in the amplitude of the MEP over time (P>0.05). (TIF) [file pone.0142857.s001.tif]

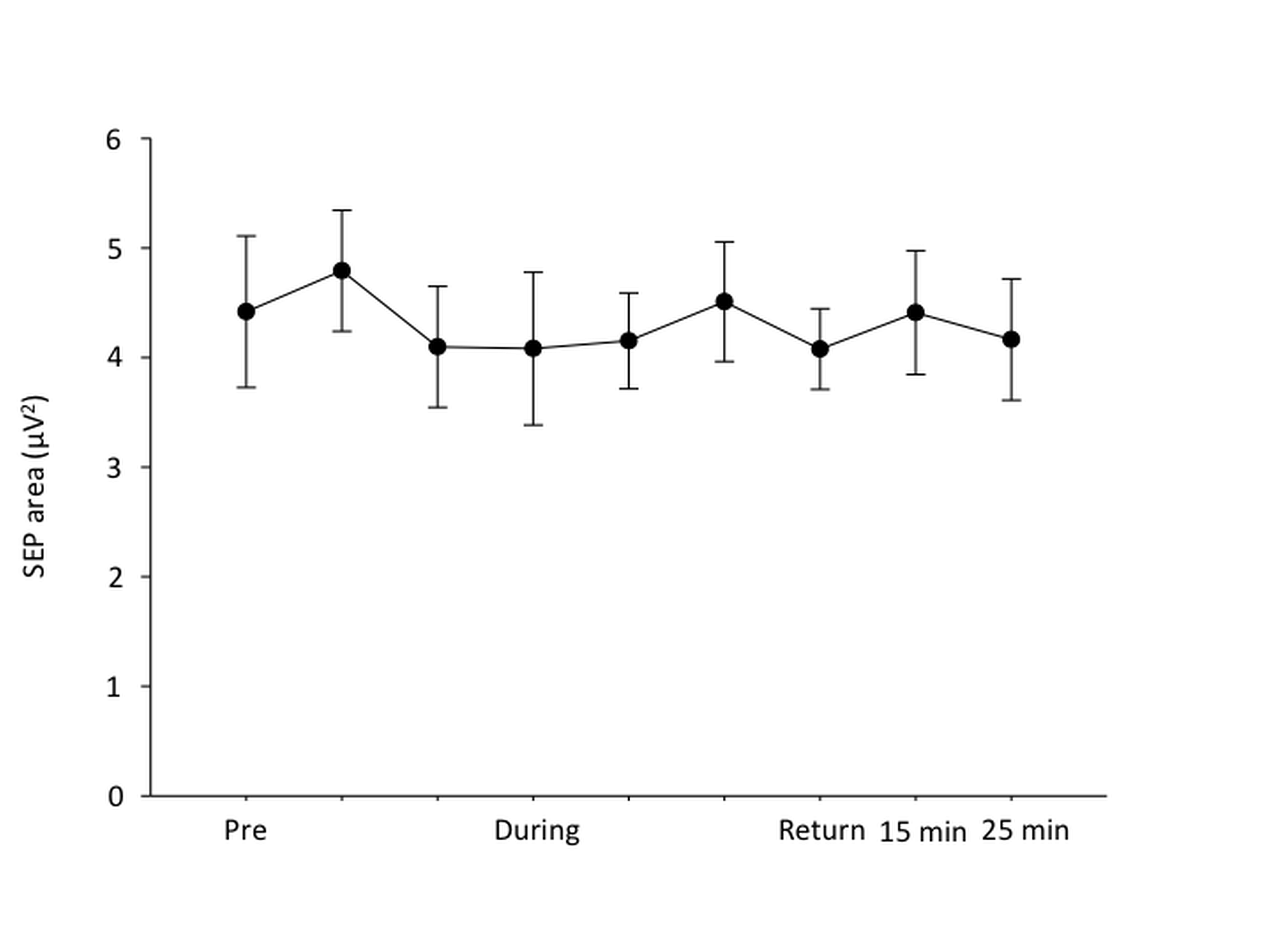

Supplement: S2 Fig — There was no change in the area of the SEP N20–P25–N33 complex over time (P>0.05). (TIF) [file pone.0142857.s002.tif]
