# Supplementary material for: New Insight into the Time-Course of Motor and Sensory System Changes in Pain
Source: PLoS One. 2015 Nov 24;10(11):e0142857. doi: 10.1371/journal.pone.0142857 (PMC4658023; doi:10.1371/journal.pone.0142857)
Supplement: S1 Table — (PDF) [file pone.0142857.s003.pdf]

S1 Table. Group data (mean and standard deviation) for amplitude and latencies of each SEP component in response to hypertonic saline infusion

SEP component	Baseline	During 1	During 2	During 3	During 4	During 5	Return to zero	15 min	25 min
Amplitude (μV)									
N9	0.086±0.069	0.089±0.071	0.089±0.07	0.093±0.077	0.075±0.066	0.074±0.064	0.092±0.083	0.094±0.078	0.094±0.087
N13	2.0±1.3	2.4±2.1	3.4±2.5	3.1±2.7	2.2±2.1	1.6±1.6	1.9±1.8	1.8±1.4	2.7±2.1
P14-N20	1.0±0.56	0.97±0.63	0.95±0.70	1.1±0.93	1.1±0.80	1.0±0.73	0.89±0.63	1.0±0.75	1.0±0.67
N20-P25	4.1±2.8	0.97±0.63	0.95±0.71	1.1±0.90	1.1±0.80	1.0±0.64	0.88±0.64	3.4±2.5	3.4±2.4
P25-N33	1.8±1.9	1.7±2.0	1.9±2.0	1.8±1.8	1.9±2.0	2.2±2.5	2.0±2.1	2.2±2.4	2.4±2.2
Latency (ms)									
N9	9.0±1.0	8.9±1.0	8.7±0.9	8.9±1.1	9.0±1.2	9.0±1.2	9.0±1.1	9.1±1.1	9.0±1.1
N13	15.7±1.1	15.7±0.9	15.3±0.8	15.4±0.9	15.6±1.0	15.7±0.8	15.8±1.0	15.8±0.9	15.6±1.0
N20	21.0±1.1	21.1±1.1	21.0±1.0	21.1±1.1	21.4±1.4	21.2±1.0	21.1±1.0	21.3±1.1	21.1±1.2
P25	31.5±3.8	30.4±4.1	31.2±4.0	31.1±3.7	31.0±4.4	31.1±3.8	31.1±3.5	31.3±3.4	30.5±3.2
N33	38.1±7.4	38.1±5.8	38.2±6.1	38.2±5.8	38.6±5.2	38.9±5.6	39.0±5.7	39.5±7.2	39.8±6.7
